# Changes in Social and Clinical Determinants of COVID-19 Outcomes Achieved by the Vaccination Program: A Nationwide Cohort Study

**DOI:** 10.3390/ijerph191912746

**Published:** 2022-10-05

**Authors:** Oliver Ibarrondo, Maíra Aguiar, Nico Stollenwerk, Rubén Blasco-Aguado, Igor Larrañaga, Joseba Bidaurrazaga, Carlo Delfin S. Estadilla, Javier Mar

**Affiliations:** 1 Osakidetza Basque Health Service, Debagoiena Integrated Health Organisation, Research Unit, 20500 Arrasate-Mondragón, Spain; 2 Biodonostia Health Research Institute, 20014 Donostia-San Sebastián, Spain; 3 Basque Center for Applied Mathematics, 48009 Bilbao, Spain; 4 Ikerbasque, Basque Foundation for Science, 48009 Bilbao, Spain; 5 Dipartimento di Matematica, Universita degli Studi di Trento, 38122 Trento, Italy; 6 Kronikgune Institute for Health Services Research, 48902 Barakaldo, Spain; 7 Public Health, Basque Health Department, 48008 Bilbao, Spain; 8 Public Health Department, University of the Basque Country, 48940 Leioa, Spain

**Keywords:** COVID-19, vaccines, infection, hospitalization, socioeconomic status, Charlson index

## Abstract

Background: The objective of this study was to assess changes in social and clinical determinants of COVID-19 outcomes associated with the first year of COVID-19 vaccination rollout in the Basque population. Methods: A retrospective study was performed using the complete database of the Basque Health Service (n = 2,343,858). We analyzed data on age, sex, socioeconomic status, the Charlson comorbidity index (CCI), hospitalization and intensive care unit (ICU) admission, and COVID-19 infection by Cox regression models and Kaplan–Meier curves. Results: Women had a higher hazard ratio (HR) of infection (1.1) and a much lower rate of hospitalization (0.7). With older age, the risk of infection fell, but the risks of hospitalization and ICU admission increased. The higher the CCI, the higher the risks of infection and hospitalization. The risk of infection was higher in high-income individuals in all periods (HR = 1.2–1.4) while their risk of hospitalization was lower in the post-vaccination period (HR = 0.451). Conclusion: Despite the lifting of many control measures during the second half of 2021, restoring human mobility patterns, the situation could not be defined as syndemic, clinical determinants seeming to have more influence than social ones on COVID-19 outcomes, both before and after vaccination program implementation.

## 1. Introduction

Analysis of the social determinants of health during the pandemic has shown significant inequalities in the incidence and severity of coronavirus disease 2019 (COVID-19) cases [1]. COVID-19 vaccines have been the core of public health strategies to control the healthcare crisis during the current pandemic [2,3], but difficulties with vaccine supply and storage have raised concerns about the equity of the distribution process [4]. Improving equity requires addressing the social determinants that lead to adverse outcomes associated with COVID-19 among the most deprived groups [5,6,7,8]. Since the severity of COVID-19 also depends on comorbidities, the assessment of inequalities must also take into account clinical variables such as comorbidities [9,10]. To help understand how COVID-19 interacts with pre-existing health inequalities, the concept of syndemic [11] has been proposed. A syndemic can be described as the biological and social interactions between conditions and states that increase individuals’ susceptibility to harm or worsen their clinical health outcomes during an epidemic [12]. The response of some countries, such as the USA, allows COVID-19 to be described as a syndemic; while under other conditions, where clinical determinants seem to have more influence on outcomes than social differences, for example, in the case of New Zealand, this definition cannot be applied [13]. Therefore, it is important for countries or regions to assess whether or not their own situation can be characterized as syndemic.

In the pre-vaccination period (2020), age, sex, and comorbidities, according to the Charlson Comorbidity Index (CCI), were identified as determinants of COVID-19 severity [14,15,16]. In addition, there has been ongoing discussion of inequalities in the incidence and severity of disease outcomes considering differences between race and ethnicity, as well as socioeconomic levels [5,7,8,17]. In an analysis conducted in 2020, it was found that clinical comorbidity was more relevant than social determinants in the outcomes associated with COVID-19 infection [8]. Nonetheless, throughout 2021, important changes have occurred due to the different responses of distinct social groups to the measures implemented to reduce social interactions and to the vaccination process [18].

Most studies of inequalities associated with COVID-19 have been performed using data aggregated at the county or local level and not with individual data due to the difficulty of integrating databases from different origins [5,6,7,19,20]. In contrast, databases based on electronic medical records (real-world data (RWD)) have been key to understanding the development of the pandemic and the effect of vaccination [21,22,23]. RWD have also been used for the analysis of inequalities in health [10,24].

The Basque Country has universal access to healthcare with a Beveridge model, providing the same healthcare in relation to COVID-19 for all inhabitants, unlike countries in which private health coverage generates inequities depending on whether individuals do or do not have coverage and the type of insurance [1]. Further, the integration of the information systems of the Basque Health Service makes it possible to study the determinants under the changing conditions of the pandemic, including, as of January 2021, the implementation of vaccination. To assess the determinants of the outcomes associated with COVID-19 infection, we need to tackle the challenge of comparing the same population at different times by the level of vaccination coverage as well as changes in confinement measures and levels of social contact [25]. Monitoring these changes is crucial to assess whether vaccination has reduced or increased inequalities related to COVID-19 in the Basque population.

The objective of this study was to assess changes in the social and clinical determinants of the outcomes related to COVID-19 in the Basque population associated with the first year of the COVID-19 vaccination program.

## 2. Materials and Methods

### Study Design and Participants

A retrospective study was carried out on the complete database of the entire organization of the Basque Health Service. Based on the Oracle Analytics System tool, which has stored, since 2003, administrative, laboratory, pharmaceutical, and clinical data, in an anonymized form, on all public health service users in all primary care centers and hospitals (outpatient clinics, emergency departments, and wards) [26,27]. In the Basque Country, access to healthcare requires a medical doctor to assign a code, based on the International Classification of Diseases (ICD), which allows a diagnosis to then be recorded in the registration system. The ICD-9-CM was used until 2015 and the ICD-10 from 2016 onwards. Previous studies have confirmed the validity of these codes [26,27]. The study protocol (ref: EOM2021029), described below, was approved by the Ethics and Clinical Research Committee of the Basque Country on 18 May 2021.

The reference population was all living individuals registered with the Basque Health Service on 1 September 2020 (2,343,858 individuals), 1 January 2021, and 1 July 2021. The following types of data were collected for each period for this entire population: age, sex, socioeconomic status (SES), diagnosis of COVID-19 infection and date, diagnoses required for the CCI with dates [14,28], and admissions to hospital and intensive care unit (ICU) facilities with dates. Age was categorized into the following groups: 0–18, 19–49, 50–65, 65 years or older. The youngest category (0–18) was selected because the vaccination process was delayed in this group. On the contrary, the oldest category (65 years or older) was prioritized during the vaccination process. The cut-off point of 50 years was established to distribute individuals of intermediate ages into two categories. Individuals with an event with the code associated with COVID-19 in the ICD-10 (U07.1) were identified as COVID-19 cases. All COVID-19 positive cases were categorized as: not requiring hospitalization or requiring hospitalization, differentiating groups based on whether or not they had required ICU admission. The date of each event (case diagnosis, hospitalization, and ICU admission) was also recorded. For the pre-vaccination period, from 1 September 2020 to 31 December 2020, we assumed a stable availability of diagnostic tests and ward and ICU beds. Moreover, we also assumed that the control measures had stabilized. In relation to the vaccination rollout process, we established three periods: pre-vaccination (September 2020–December 2020), initial vaccination (January 2021–June 2021), and advanced vaccination (June 2021–December 2021). The vaccination program began in January 2021, reaching vaccination coverage for the population over 12 years of age as follows: 6.0% in March, 47.2% in June, 88.9% in September, and 90.9% in December, with 44.7% having received a booster dose by the end of the year [29].

The CCI [28] quantifies the mortality risk associated with 19 weighted comorbidities. This index has been reported to be a significant prognostic factor in patients with COVID-19 [14].

As an indicator of SES, we used the individual’s pharmacy co-payment code that is based on the household’s income. Specifically, the Basque Health Service assigns this code to individuals based on their income tax returns. Previous studies have provided evidence of its validity [30]. The low-income category (“low SES”) included pensioners with a non-contributory pension, disabled individuals, and unemployed workers who had exhausted their unemployment benefit. The medium-income category (“medium SES”) included individuals with an income (workers or pensioners with a contributory pension) under €18,000, and the high-income category (“high SES”) those with an income (workers or pensioners with contributory pension) equal to or greater than €18,000.

## 3. Statistical Analysis

We used the statistical software R (version 4.1.3) to perform the statistical analysis, and the significance threshold was set at *p* < 0.05. First, a descriptive analysis of the entire population was performed in each period according to individuals’ infection status during the period. In a second step, a multivariate analysis was performed using Cox regression models to measure the risks (hazard ratios) of infection, hospitalization, and admission to the ICU adjusted for age, sex, CCI, and SES [31,32]. The Cox proportional hazards model assumption was tested by checking Schoenfeld residuals. We built models for each period (2020, first half of 2021, and second half of 2021) separately to analyze the changes in the determinants of the COVID-19-related outcomes and a model with the period as a covariate. Finally, Kaplan–Meier curves were constructed comparing the risks of infection and hospital and ICU admission in the three periods according to SES and CCI, and the differences were analyzed using log-rank tests.

## 4. Results

Table 1 shows the sociodemographic (age, sex, and income level) and clinical determinants (CCI) in the Basque population (2,343,858 in 2020) in the three periods analyzed according to whether or not individuals were infected during follow-up. The percentages of positive cases were 3.1% in the last quarter of 2020, 3.6% in the first half, and 6.0% in the second half of 2021. In the three periods, the younger age groups had a higher risk of infection with a maximum of 8.2% among 18- to 50-year-olds in the second half of 2021 and a minimum of 2.4% in over-65-year-olds in 2020. Table 2 of the supplementary material disaggregates vaccination coverage by SES in the third period. Notably, the percentage of vaccination was higher in individuals with high SES (72.4%) than those with low SES (70.4%).

Table 3 contains the results for each period of the multivariate regression analyses with Cox models of the risk of infection for the entire population and risks of hospital and ICU admission for those infected. Women had a significantly higher risk of becoming infected and much lower risks of hospital and ICU admission in all three periods. With older age, the risk of infection fell significantly, but the risks of hospital and ICU admission increased. The classification by CCI followed what was expected in all periods, with moderate increases in the risk of infection and large increases in the risks of hospital and ICU admission with greater comorbidity. The income level (SES) followed different trends across the periods. In individuals with higher incomes, the risk of infection was higher in all three periods and increased even more in the second half of 2021. In contrast, no significant differences were detected in the risk of hospitalization by SES in the first two periods, though it was significantly lower for the high SES group in the third period. Differences in the risk of admission to the ICU by SES only reached significance in the third period, in which the group with higher income had a lower risk.

The models showing the association of the three risks analyzed with the three periods adjusted for sex, age, comorbidity, and income level are reported in Table 4**.** Comparing the first period (without vaccination) with the third (advanced vaccination), the risk of infection was somewhat higher in the third, and the most striking finding is the much lower severity of cases in this last period, evidenced by the HR of 0.451 for hospitalization and 0.457 for ICU admission.

**Figure 1:**
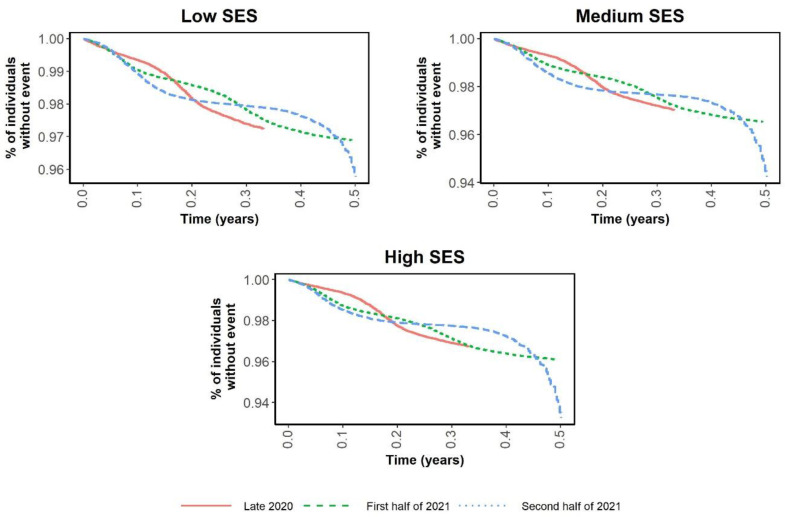
Kaplan–Meier curves of time to infection by socioeconomic level and period.

**Figure 2:**
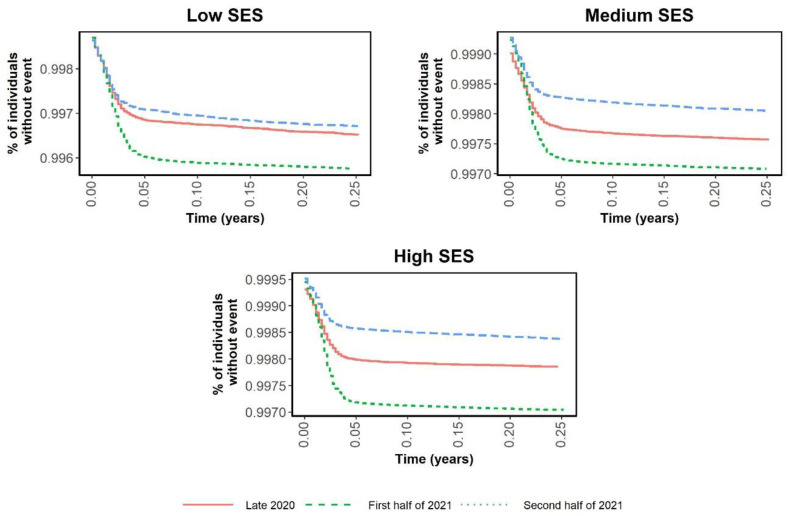
Kaplan–Meier curves of time to hospitalization according to socioeconomic level and period.

**Figure 3:**
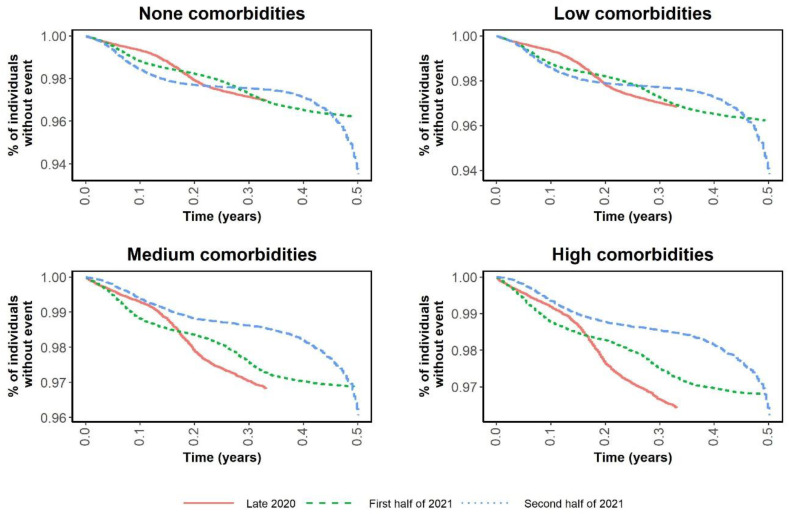
Kaplan–Meier curves of time to infection by Charlson Comorbidity Index and period.

**Figure 4:**
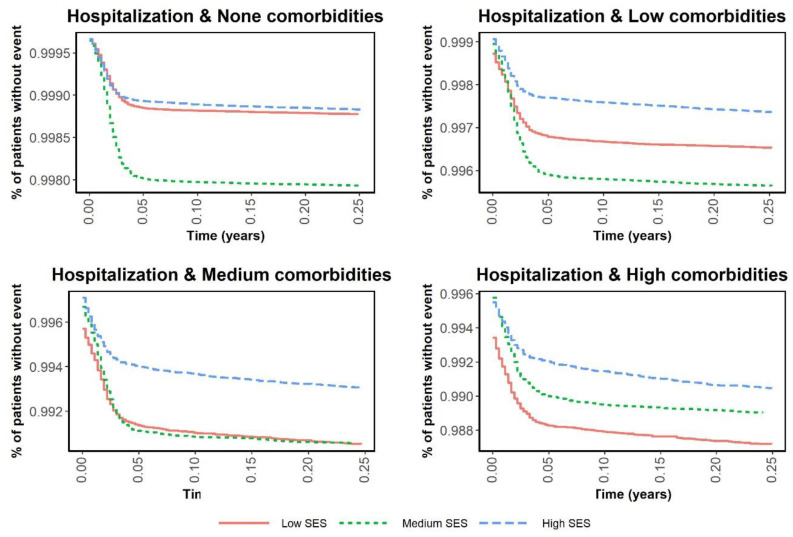
Kaplan–Meier curves of time to hospitalization by Charlson Comorbidity Index and period.

Figure 1 and Figure 2 are Kaplan–Meier curves comparing the risks of infection and hospitalization in the three periods according to SES. The curves in Figure 1 intertwine as the incidence rates change in each period, while Figure 2 shows that the risk of hospitalization was notably lower in the medium and high SES groups. Figure 3 and Figure 4 show the same Kaplan–Meier curves disaggregated by CCI. While individuals with no comorbidities or a low CCI had similar COVID-19-related outcomes in the three periods, in the groups with greater comorbidity (CCI ≥ 3) the risk of hospitalization was lower when the vaccination program had been fully implemented.

## 5. Discussion

The vaccination program in the Basque Country produced lower changes in the socioeconomic than the clinical determinants of the outcomes associated with COVID-19. Comorbidity levels measured by the CCI were consistently associated with risks in all three time periods considered, CCI being the main driver of hospitalization. On the contrary, SES was only a significant variable for hospitalization in the third period. In all groups, the vaccination rollout did not eliminate the risk of infection, but it greatly reduced the use of resources since the severity of the condition was much lower and individuals were much less likely to require hospitalization. The most striking effect related to determinants was that the risk of hospitalization was halved (HR = 0.507) in the third period for the highest income group, while in the first two periods there were no statistically significant differences by SES in hospital admissions. When using the period as an adjustment variable in the model, the difference was smaller, but it was still relevant and statistically significant. Contrarily, from the period before vaccination (2020) to that of complete vaccination (second half of 2021), the HR for infection increased for high-income individuals. Comorbidity behaved in the same way over time, the risk of infection and hospitalization increasing in all three periods in parallel with the CCI categories.

The probability of infection can be expressed as the combination of an absolute risk due to the environment and a relative risk depending on the characteristics and decisions of the individual [33]. Comparing the periods in this study, we are jointly taking into account the absolute risk associated with the predominant variant of the virus (alpha in the first half of 2021 and delta in the second), the measures of home confinement and social mobility restriction, the policies obliging use of masks, and the deployment of the vaccination program. Similarly, the analysis of the determinants indicates that each individual has a relative risk that is associated with age, sex, comorbidity (CCI), SES, and the individual response to the offer of vaccination. The finding that COVID-19 spread faster in the least deprived group and that it even increased when vaccination rollout achieved higher coverage is only explained by a more active social life, increasing the number of contacts with people outside the family circle. We propose two possible reasons for this. First, a higher income facilitates socializing, this being associated with consumption in bars and restaurants in the 18- to 45-year-old age group, which was the one with the highest incidence in the third period. Second, a better knowledge of the effects of the vaccine in this group mitigated the fear of COVID-19, which translated into greater mobility and, consequently, a higher probability of infection. Although, in general, deprivation and therefore lower SES have been associated with a greater spread of COVID-19 [1,6]; some studies involving geographical analyses have indicated that the choice of the time interval contributes to a lack of consistency with which socioeconomic variables are found to be relevant in the analysis of COVID-19-related outcomes [20]. In a county-level spatial analysis in the USA, Boland et al. found that the risk of COVID-19 infection was lower in counties with high rates of public assistance use despite those counties having higher levels of poverty [19]. As in our results, the pattern could be explained by lower social mobility and its direct relationship with the risk of infection [20]. A modelling study conducted with Basque data also found that vaccines can boost infections because of their effect on reducing symptoms but not stopping virus transmission [34].

The reduction in the hospitalization HR in the third period in the high-income group could be explained by higher vaccination coverage. Although vaccination was a universal program with centrally managed delivery by the Basque Health Service, its voluntary nature caused differences in its acceptance by different socioeconomic groups. Similar findings have been reported in other countries, including the USA [35,36]. Our classification of income level identified pensioners with a non-contributory pension, disabled individuals, and workers who had exhausted their unemployment benefit as having a low SES. These people make up the 10% of the population with the greatest deprivation and therefore with the greatest social and cultural barriers to accessing the program. Another factor that influences the lower hospitalization rate observed is the fact that 20% of the Basque population has double healthcare coverage (public and private). Private providers are mostly used by high-income individuals, and this could have led to an underreporting of hospitalizations due to the non-inclusion of private hospital admissions in our database. Nonetheless, this would not explain the lack of differences in 2020 and the first half of 2021.

Part of the differences in findings concerning the determinants of COVID-19 outcomes is due to the designs and data sources differing between studies [20]. While several studies have indicated differences in the acceptance of COVID-19 vaccine programs between countries according to their income level and between geographic areas within each country [37,38], the analysis of individual data provides a better understanding of socioeconomic inequalities associated with COVID-19 [8]. In Denmark, social deprivation as well as psychiatric and medical disorders have been associated with a lower vaccination rate for COVID-19 [39]. On the other hand, in other research at county level in the USA, the rate of public assistance use and the level of physical exercise were identified as protective factors against mortality [19,20].

The main strength of our study is its design as a region-wide study based on a population database with information at the individual level on socioeconomic status and comorbidities. The validity of the diagnoses has been previously documented [26]. In addition, the level of household income was assigned using individuals’ co-payment categories, which are based on employment and income information from government tax records, and previous studies have provided evidence of the validity of this approach [30]. Statistical analyses with individual data provide better evidence than geographic or spatial analyses because they maintain the correlation of variables for each person. As weaknesses, we should mention the lack of data on private hospitalizations [40] and the failure to consider other socioeconomic variables such as educational level [20].

## 6. Conclusions

The conclusion of our findings is that, in the changing context of the COVID-19 pandemic, the vaccination program met the objective of reducing the epidemiological impact even though mobility restrictions were much less strict during the second half of 2021. At the same time, the only inequality identified was the lower probability of hospitalization in the high-income group; hence, COVID-19 in the Basque population could not be characterized as syndemic [11,13]. The use of RWD has made it possible to evaluate the impact of health policies in terms of reducing inequalities. Despite the fact that control measures were lifted during the second half of 2021, restoring human mobility patterns, the situation in the Basque population could not be defined as a syndemic, clinical determinants seeming to have more influence than social ones on COVID-19-related outcomes, before and after vaccination program implementation.

## Figures and Tables

**Table 1:** Characteristics of infected and uninfected individuals during late 2020 and the first and second halves of 2021. Charlson: Charlson Comorbidity Index.

	September 2020 to December 2020		January 2021 to June 2021		July 2021 to December 2021	
	Infected	Not Infected	Infected	Not Infected	Infected	Not Infected
Men	34,210 (2.98%)	1,112,476 (97.02%)	41,959 (3.67%)	1,100,499 (96.33%)	68,148 (6.00%)	1,068,389 (94.00%)
Women	38,244 (3.19%)	1,158,928 (96.81%)	43,155 (3.62%)	1,149,882 (96.38%)	72,195 (6.08%)	1,114,933 (93.92%)
Age <18 years	7932 (4.14%)	183,797 (95.86%)	10,546 (5.73%)	173,353 (94.27%)	13,586 (7.90%)	158,459 (92.10%)
Age 18–50 years	33,916 (3.29%)	998,477 (96.71%)	42,056 (4.10%)	984,330 (95.90%)	83,488 (8.22%)	932,597 (91.78%)
Age 50–65 years	16,912 (3.01%)	544,245 (96.99%)	19,330 (3.43%)	545,017 (96.57%)	27,610 (4.85%)	541,683 (95.15%)
Age ≥65 years	13,694 (2.45%)	544,885 (97.55%)	13,182 (2.35%)	547,681 (97.65%)	15,659 (2.77%)	550,583 (97.23%)
Low income	6679 (2.77%)	234,797 (97.23%)	7501 (3.11%)	233,506 (96.89%)	10,116 (4.23%)	229,011 (95.77%)
Medium income	32,128 (2.99%)	1,043,585 (97.01%)	37,325 (3.49%)	1,033,278 (96.51%)	61,386 (5.77%)	1,003,396 (94.23%)
High income	33,647 (3.28%)	993,022 (96.72%)	40,288 (3.93%)	983,597 (96.07%)	68,841 (6.75%)	950,915 (93.25%)
Charlson 0	49,275 (3.04%)	1,572,181 (96.96%)	59,542 (3.81%)	1,503,142 (96.19%)	100,376 (6.49%)	1,447,323 (93.51%)
Charlson 1–2	17,834 (3.17%)	543,993 (96.83%)	20,627 (3.78%)	525,009 (96.22%)	33,799 (6.17%)	513,829 (93.83%)
Charlson 3–4	3187 (3.18%)	96,929 (96.82%)	30,490 (3.13%)	94,462 (96.87%)	3890 (3.94%)	94,915 (96.06%)
Charlson >4	2158 (3.57%)	58,301 (96.43%)	18,950 (3.20%)	57,266 (96.80%)	2277 (3.77%)	58,093 (96.23%)
Total	72,454 (3.1%)	2,271,404 (96.9%)	85,114 (3.6%)	2,250,381 (96.4%)	140,343 (6.0%)	2,183,322 (94.0%)

**Table 2:** Distribution of vaccination by socioeconomic level and period.

	Full Vaccination as of 1 July 2021			
	Unvaccinated		Vaccinated	
Low income	69,089	29.6%	164,355	70.4%
Medium income	375,741	35.9%	671,005	64.1%
High income	273,287	27.6%	717,964	72.4%
Total	718,117	31.6%	1,553,324	68.4%

**Table 3:** Change in the adjusted risk of cases, hospitalizations, and ICU admissions in the three periods measured by hazard ratios (Cox regression).

	2020-09 to 2020-12			2021-01 to 2021-06			2021-07 to 2021-12		
Infection	HR	Lower CI	Upper CI	HR	Lower CI	Upper CI	HR	Lower CI	Upper CI
Man	Ref.			Ref.			Ref.		
Woman	1.114 ***	1.098	1.13	1.022 **	1.009	1.036	1.073 ***	1.061	1.084
Age	0.991 ***	0.99	0.991	0.986 ***	0.986	0.987	0.978 ***	0.977	0.978
Low income	Ref.			Ref.			Ref.		
Medium income	1.048 ***	1.021	1.076	1.047 ***	1.021	1.073	1.215 ***	1.19	1.241
High income	1.179 ***	1.148	1.211	1.203 ***	1.174	1.233	1.472 ***	1.441	1.503
Charlson 0	Ref.			Ref.			Ref.		
Charlson 1–2	1.138 ***	1.118	1.158	1.118 ***	1.100	1.136	1.138 ***	1.124	1.153
Charlson 3–4	1.380 ***	1.33	1.433	1.237 ***	1.191	1.284	1.178 ***	1.14	1.218
Charlson >4	1.553 ***	1.486	1.624	1.267 ***	1.209	1.328	1.134 ***	1.086	1.183
Hospitalization	HR	Lower CI	Upper CI	HR	Lower CI	Upper CI	HR	Lower CI	Upper CI
Man	Ref.			Ref.			Ref.		
Woman	0.667 ***	0.627	0.709	0.674 ***	0.631	0.721	0.718 ***	0.655	0.787
Age	1.044 ***	1.042	1.046	1041 ***	1.039	1.043	1.016 ***	1.013	1.018
Low income	Ref.			Ref.			Ref.		
Medium income	1.028	0.939	1.126	0.951	0.863	1.049	0.739 ***	0.654	0.835
High income	0.978	0.891	1.074	0.999	0.904	1.103	0.507 ***	0.444	0.579
Charlson 0	Ref.			Ref.			Ref.		
Charlson 1–2	1.759 ***	1.634	1.894	1.533 ***	1.418	1.657	1.489 ***	1.332	1.663
Charlson 3–4	2.706 ***	2.458	2.977	2.161 ***	1.944	2.402	3.097 ***	2.665	3.598
Charlson >4	3.551 ***	3.203	3.937	2.597 ***	2.309	2.922	4.226 ***	3.609	4.95
ICU admission	HR	Lower CI	Upper CI	HR	Lower CI	Upper CI	HR	Lower CI	Upper CI
Man	Ref.			Ref.			Ref.		
Woman	0.339 ***	0.27	0.424	0.426 ***	0.341	0.531	0.512 ***	0.375	0.7
Age	1.028 ***	1.023	1.034	1.025 ***	1.019	1.031	1.005	0.997	1.013
Low income	Ref.			Ref.			Ref.		
Medium income	1.042	0.749	1.451	1.039	0.739	1.461	0.794	0.513	1.229
High income	1.059	0.760	1.476	1.162	0.828	1.63	0.550 *	0.347	0.872
Charlson 0	Ref.			Ref.			Ref.		
Charlson 1–2	1.735 ***	1.376	2.187	2.120 ***	1.676	2.682	1.545 *	1.096	2.178
Charlson 3–4	2.135 ***	1.512	3.015	2.528 ***	1.773	3.605	2.563 ***	1.494	4.396
Charlson >4	2.373 ***	1.597	3.524	2.445 ***	1.595	3.747	1.704	0.801	3.628

HR: hazard ratio; CI: 95% confidence interval; * *p* < 0.05; ** *p* < 0.01; *** *p* < 0.001; ICU: intensive care unit; Charlson: Charlson Comorbidity Index.

**Table 4:** Change in the adjusted risk of cases, hospitalizations, and ICU admissions in the three periods measured by hazard ratio (Cox regression) including the period as a covariate.

	Infection			Hospitalization			ICU Admission		
	HR	Lower CI	Upper CI	HR	Lower CI	Upper CI	HR	Lower CI	Upper CI
Period 2020	Ref.			Ref.			Ref.		
Period 2021-1	0.705 ***	0.698	0.713	0.886 ***	0.848	0.925	0.987	0.857	1.136
Period 2021-2	1.085 ***	1.074	1.096	0.451 ***	0.427	0.476	0.457 ***	0.383	0.546
Man	Ref.			Ref.			Ref.		
Woman	1.068 ***	1.061	1.076	0.680 ***	0.653	0.708	0.402 ***	0.35	0.463
Age	0.983 ***	0.983	0.984	1.037 ***	1.036	1.038	1.022***	1.019	1.026
Low income	Ref.			Ref.			Ref.		
Medium income	1.115 ***	1.1	1.13	0.936 *	0.883	0.992	0.988	0.802	1.217
High income	1.306 ***	1.288	1.324	0.866 ***	0.815	0.92	0.972	0.788	1.199
Charlson 0	Ref.			Ref.			Ref.		
Charlson 1–2	1.134 ***	1.124	1.144	1.621 ***	1.545	1.702	1.844 ***	1.59	2.139
Charlson 3–4	1.259 ***	1.233	1.286	2.544 ***	2.386	2.713	2.353 ***	1.88	2.946
Charlson >4	1.297 ***	1.264	1.331	3.264 ***	3.045	3.499	2.297 ***	1.754	3.008

HR: hazard ratio; CI: 95% confidence interval; * *p* < 0.05; ** *p* < 0.01; *** *p* < 0.001; ICU: intensive care unit; Charlson: Charlson Comorbidity Index.

## Data Availability

Data were provided by the Basque Health Service. Our data sharing agreement clearly stipulates that they cannot be shared with any third party.
